# Templated Assembly of pH-Labile Covalent Organic Framework Hierarchical Particles for Intracellular Drug Delivery

**DOI:** 10.3390/nano12173055

**Published:** 2022-09-02

**Authors:** Fangzhou Zhou, Yuanyuan Fang, Chao Deng, Qian Zhang, Minying Wu, Hsin-Hui Shen, Yi Tang, Yajun Wang

**Affiliations:** 1 Department of Chemistry, Fudan University, Shanghai 200433, China; 2 College of Chemistry & Materials Engineering, Wenzhou University, Wenzhou 325027, China; 3 Department of Materials Science and Engineering, Monash University, Clayton, VIC 3800, Australia

**Keywords:** mesoporous silica, covalent organic framework, hierarchically porous, templated assembly, anticancer, drug delivery

## Abstract

Covalent organic frameworks (COF), a class of emerging microporous polymers, have been restrained for drug delivery applications due to their limited controllability over particle sizes and degradability. Herein, a dendritic mesoporous silica nanosphere (DMSN)-mediated growth strategy is proposed to fabricate hierarchical DMSN@COF hybrids through in situ growing of 1,3,5-tris(4-aminophenyl)benzene and 2,5-dimethoxyterephthaldehyde connected COF with acid cleavable C=N bonds. After the removal of the DMSN template, COF hierarchical particles (COF HP) with tailored particle sizes and degradability were obtained. Notably, the COF HP could be degraded by 55% after 24 h of incubation at pH 5.5, whereas the counterpart bulk COF only showed 15% of degradation in the same conditions. Due to the improved porosity and surface area, the COF HP can be utilized to load the chemotherapeutic drug, doxorubicin (DOX), with a high loading (46.8 wt%), outperforming the bulk COF (32.1 wt%). Moreover, around 90% of the loaded DOX can be discharged from the COF HP within 8 h of incubation at pH 5.5, whereas, only ~55% of the loaded DOX was released from the bulk COF. Cell experiments demonstrated that the IC_50_ value of the DOX loaded in COF HP was 2–3 times lower than that of the DOX loaded in the bulk COF and the hybrid DMSN@COF. Attributed to the high loading capacity and more pH-labile particle deconstruction properties, COF HP shows great potential in the application as vehicles for drug delivery.

## 1. Introduction

Covalent organic framework (COF), a family of emerging porous crystalline polymers, has a periodic network structure connected by covalent bonds [1,2]. Due to their distinctive properties such as high surface area, ordered pore structure, abundant functional groups, and good stability, COF materials have gained increasing interest in a range of applications, ranging from catalysis [3,4], molecular separation [5,6], energy storage [7,8], and sensing [9,10] to drug delivery [11,12]. As for drug delivery applications, it is important to design materials with biocompatible, biodegradable, and stimuli-responsive release properties as well as high loading capacities. To achieve stimuli-responsive release properties, the researchers developed a class of COF materials connected via acid degradable covalent bonds, which makes them promising as intracellular drug delivery vehicles [13,14,15,16,17,18]. For instance, Liu et al. [19] reported the 1,3,5-tris(4-aminophenyl)benzene (TAPB) and 2,5-dimethoxyterephthaldehyde (DMTP) connected COF (TAPB-DMTP COF) with cleavable C=N bonds under an acidic environment, which allows for the deconstruction of COF materials inside the subcellular compartments (e.g., endosomes, lysosomes) of tumor cells with an acidic condition of ca. pH 5.5.

Nevertheless, COF materials are generally irregular in shape and difficult to control over the particle size, hence restricting their performance in drug delivery applications [14,15,20]. Additionally, COF materials comprise of highly stacked micropores, resulting in reduced mass transfer efficiency and lower drug loading efficiency [13,20,21]. Therefore, a tailored synthesis of COF materials with a uniform particle size and hierarchical porosity is of importance in improving the performance of COF materials in the drug delivery field. Template synthesis is a powerful technique for preparing materials with well-defined nanoarchitectures for a wide range of practical applications [22,23]. To date, a variety of substrates such as magnetic nanoparticles [24], graphene [25], MXenes [26], SiO_2_ nanoparticles [21,27], Al_2_O_3_ ceramic [28], diatomite [29], and ZnO nanorods [30] have been applied for preparing COF materials with various architectures.

Mesoporous silicas have attracted extensive interest due to their high surface area, good biocompatibility, highly tunable morphology, and pore structures. To date, mesoporous silicas of various shapes, ranging from nanospheres [31,32,33], hollow spheres [34,35,36], nanobottles [37], nanorods [38,39,40] to nanotubes [41,42] have been prepared. Among them, a novel type of mesoporous silica nanoparticle with a dendritic pore structure (denoted as DMSN) is particularly attractive due to its various favorable physical properties such as uniform and small particle size, high pore volume, large pore size, and a highly accessible, central-radial pore structure [35,43,44].

**Figure 1:**
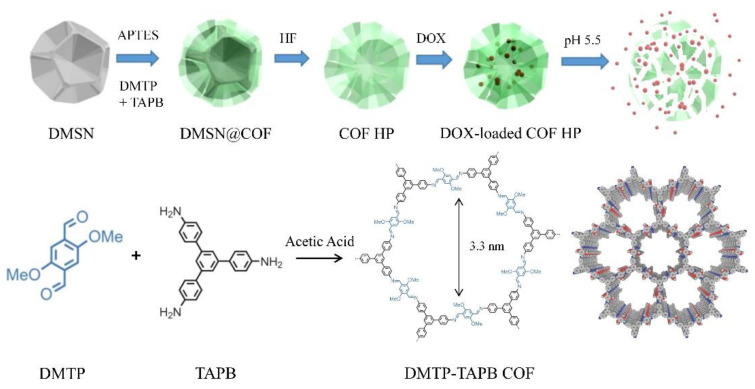
A schematic diagram of the preparation of COF HP and the delivery of DOX.

Herein, we investigated the application of DMSN as the sacrificial template for the preparation of TAPB-DMTP COF particles with hierarchical porosity (COF HP) (Figure 1). The COF HP was synthesized by an in situ condensation reaction between the TAPB and DMTP monomers on the DMSN template. The thickness of the COF layer could be tuned by changing the monomer concentrations in the synthesis. After removal of the DMSN template, highly crystalline COF HP replicas with a hierarchical pore structure, tailored particle size, excellent colloidal stability, and pH-responsive degradation properties were obtained. The unique hierarchical pore structure not only improves the particle’s loading capacity, but also the degradation ability of COF HP, allowing for intracellular delivery of the chemotherapeutic drug (i.e., doxorubicin) more efficiently.

## 2. Materials and Methods

### 2.1. Materials

Tetraethyl orthosilicate (TEOS), cetyl-trimethylammonium tosylate (CTATos), 3-aminopropyltriethoxysilane (APTES), and triethanolamine were obtained from Sigma-Aldrich (St. Louis, MO, USA). 2,5-Dimethoxyterephthaldehyde (DMTP) and 1,3,5-tris(4-aminophenyl)benzene (TAPB) were obtained from TCI (Shanghai, China). Acetic acid, hydrofluoric acid (40%), ammonium hydroxide solution (25 wt%), methanol, ethanol, acetonitrile, acetone, dimethyl sulfoxide (DMSO), and N-dimethylformamide (DMF) were purchased from Sinopharm Chemical Reagent Co. Ltd. Doxorubicin (DOX) and 3-(4,5-dimethylthiazol-2-yl)-2,5-diphenyltetrazolium bromide (MTT) were obtained from Shanghai Aladdin Bio-Chem Technology Co. Ltd. Dullbecco’s modified Eagle’s medium (DMEM) and 10% fetal bovine serum were purchased from Gibco (Shanghai, China). Deionized water was prepared by an ultrapure water treatment system (Barnstead™ GenPure™ Thermo Scientific™). All reagents used in the experiment were of analytical grade without further purification.

### 2.2. Synthesis of the DMSN

DMSN was prepared based on a reported method [32]. In brief, 1.92 g of CTATos and 0.347 g of triethanolamine was dissolved in 100 mL of deionized water at 80 °C for 1 h. Then, 14.58 g of TEOS was dropwise added and the mixture was stirred for 2 h. The produced DMSN was centrifuged (8000 rpm, 10 min) and washed twice with ethanol and water. After drying at 60 °C for 12 h, the product was calcined at 550 °C for 6 h in air to remove the surfactant from DMSN. To modify the particle with amine groups, 1.0 g of DMSN was dispersed in 50 mL of ethanol, and then added with 1 mL of ammonia solution (25 wt%) and 1 mL of APTES. After stirring at 25 °C for 3 h, the product was centrifuged and washed three times with a water/ethanol solution (50%/50%, *v*/*v*) to obtain the amine-modified DMSN (DMSN-NH_2_).

### 2.3. Synthesis of the DMSN@COF and COF HP

First, 18 mg of DMSN-NH_2_ was dispersed in 100 mL of acetonitrile, then added with 0.8 mg of DMTP (0.0048 mmoL) and 1 mg of TAPB (0.0028 mmoL) under brief sonication (5 min). After adding 0.05 mL of acetic acids, the mixture was agitated at room temperature for 24 h,. Finally, the solids were centrifuged (8000 rpm, 10 min), washed twice with methanol and acetone, and then dried at 60 °C for 12 h to yield a hybrid DMSN@COF. By changing the amount of DMTP and TAPB monomers, DMSN@COF with different COF contents were obtained. Three more DMSN@COF samples with the DMTP/TAPB dosage of 2.4 mg/3 mg, 4 mg/5 mg, 8 mg/10 mg, and an equal proportion of acetic acid were also prepared. For convenience, samples prepared with the above four DMTP/TAPB dosages were denoted as DMSN@COF-0.1, DMSN@COF-0.2, DMSN@COF-0.5, and DMSN@COF-1.0, respectively. To remove the DMSN template, 1 mg of DMSN@COF was dispersed in 1 mL of a 1:1 ethanol/water solution, and added to 100 μL of 5 M HF. After 5 min of agitation, the product was collected by centrifugation (8000 rpm, 10 min) and washed with water three times. The four COF HP samples obtained from DMSN@COF prepared with different DMTP/TAPB dosages were denoted as COF HP-0.1, COF HP-0.3, COF HP-0.5, and COF HP-1.0, respectively.

### 2.4. Synthesis of the Bulk COF

In a typical synthesis, 8 mg of DMTP (0.048 mmoL) and 10 mg of TAPB (0.028 mmoL) were diluted in 10 mL of acetonitrile and then mixed. After adding 0.5 mL of acetic acid, the mixture was agitated for 24 h at room temperature. The precipitate was collected by centrifugation and washed with DMF and acetone three times. The collected powder was dried under vacuum for 12 h.

### 2.5. Degradation Property of the Nanocarriers

To investigate the degradation properties of the nanocarriers, the DMSN, bulk COF, and COF HP samples were dispersed in pH 5.5 phosphate buffers with a solid concentration of 0.1 mg/mL. The mixture was incubated at 37 °C, and the turbidity of the suspension was monitored by DLS at different time intervals. The relative turbidity was calculated as the ratio of the scattering intensity of nanocarriers to that of the initial turbidity.

### 2.6. DOX Loading and Release

To load the particles with DOX, 2 mg of COF-based nanocarriers was mixed with 5 mL of DOX (1 mg/mL) in PBS buffer (pH 7.4). After stirring for 6 h, the free DOX was removed by centrifugation at 8000 rpm for 10 min. The DOX in the supernatant was measured via UV–Vis spectrophotometer at 480 nm using an established calibration curve. The mass of DOX loaded in the nanocarriers was calculated by subtracting the mass of DOX in the supernatant from the initially added drug. To study the DOX release properties, DOX-loaded nanocarriers were dispersed in 3 mL of PBS solution of different pH (7.4 and 5.5, respectively) and gently shaken at 37 °C. At different intervals, the suspension was centrifuged (8000 rpm, 10 min) and the supernatant was removed for UV–Vis measurement. Fresh PBS solution was then added to continue the release experiments. The DOX release data were averaged with three measurements. The normalized release was calculated as the ratio of the amount of DOX determined in the release medium to that of the drug loaded in the nanocarriers.

### 2.7. MTT Assay

The cytotoxicity assay against L929 cells (mouse fibroblast cell) and A549 cells (human adenocarcinoma alveolar basal epithelial cell) was evaluated by the standard 3-(4,5-dimethylthiazol-2-yl)-2,5-diphenyltetrazolium bromide (MTT) assay. The cells were cultured in Dullbecco’s modified Eagle’s medium (DMEM) with 10% fetal bovine serum in a humidified atmosphere with 5% CO_2_ at 37 °C. The cells were incubated for 24 h in 96-well plates at a density of 6 × 10^3^ cells/well. Then, the L929 cells were treated with DMSN, DMSN@COF, COF HPs, or bulk COF, while the A549 cells were treated with free DOX, DOX-loaded nanocarriers, or blank nanocarriers at a series of concentrations for 24 h at 37 °C. After that, the medium was replaced with a DMEM solution containing 10 μL of MTT (5 mg/mL), and the cells were incubated for 4 h in the dark. After the removal of the supernatant, 150 μL of DMSO was added to dissolve the formazan crystals generated by live cells. The absorbance at 490 nm was monitored by a microplate reader, and the data were averaged from measurements in five wells. The relative cell viability was determined by comparing the absorbance from the treated cells to that of the control cells, which were incubated in the cell culture media only.

### 2.8. Characterization

A Zeiss Gemini 300 scanning electron microscope (SEM) operating at 5 kV and an FEI Tecnai G2 transmission electron microscope (TEM) operating at 200 kV were used to characterize the morphology of the materials. The X-ray diffraction (XRD) analyses were performed on a PANanlytical X’Pert PRO MRD diffractometer operating at 45 kV and 40 mA. A Nicolet 6700 spectrometer was used to record the Fourier transform infrared (FTIR) spectra in KBr plate. The N_2_ sorption isotherms of the materials were measured on an ASAP 2020 Plus surface area and pore size analyzer (Micromeritics, Norcross, GA, USA) at 77 K. The samples were degassed by heating at 120 °C (heating rate: 5 °C/min, dwell time: 12 h). The pore size distributions were estimated using the quenched-solid density functional theory (QSDFT) model for slit/cylindrical pores (adsorption branch; N_2_ at 77 K on carbon). The particle size was measured by dynamic light scattering (DLS, Zetasizer Nano ZS90, Malvern Instruments Ltd., Malvern, UK). The absorption spectra were recorded on a UV 2600 UV–Vis–NIR spectrophotometer (Shimadzu, Japan) with 1 cm path-length cells at a wavelength of 480 nm. MTT experiments were carried out using a microplate reader (Thermo Multiskan skyhigh).

## 3. Results and Discussion

Figure 1 depicts the strategy used for preparing the DMSN@COF hybrids and COF HP replicas. Monodispersed DMSN were used as the porous template. To activate the particle surface with amine groups, modification of the DMSN with APTES was performed via a convenient silane chemistry. Following a published protocol with minor modification for COF DMTP-TAPB synthesis [45,46], the amine group grafted DMSNs were employed for the in situ growth of a COF coating, thereafter, obtaining the hybrid DMSN@COF. The DMTP-TAPB COF chosen as its structure is pH-labile in a weak acidic environment (e.g., pH 5.5), a microenvironment of tumor cells [19,21]. After the removal of the DMSN template, the COF HP replicas were obtained. Due to the high porosity of COF HP, DOX can be efficiently loaded in the nanocarriers. Importantly, the unique hierarchical pore structure of COF HP increases the contact area and diffusion rate between the solution and the COF material, thereby a higher degradation ability of the COF, which allows for a faster release of the loaded DOX in tumor cells.

**Figure 2:**
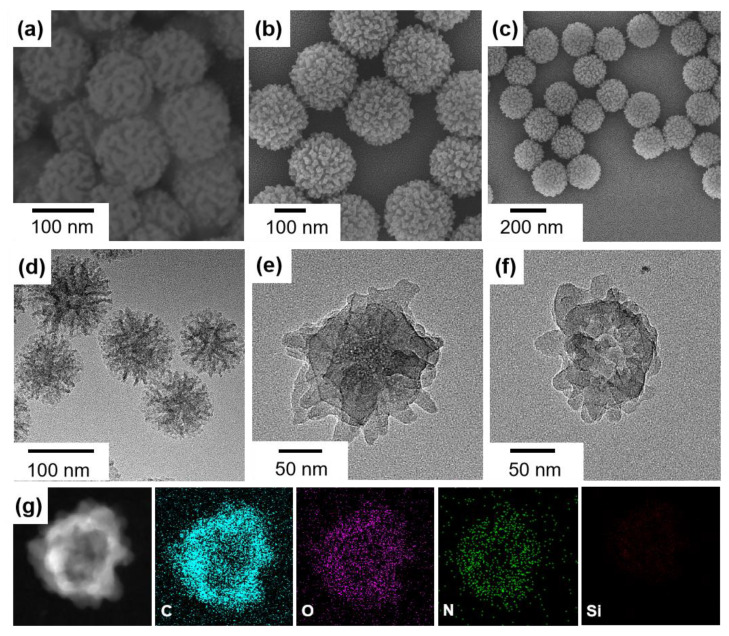
The SEM (**a**–**c**) and TEM (**d**–**f**) images of the DMSN (**a**,**d**), DMSN@COF (**b**,**e**), and COF HP (**c**,**f**). The EDX elemental mapping of COF HP (**g**).

SEM and TEM were used to characterize the morphology of the materials. The original DMSN template was ca. 100 nm in diameter with a dendritic channel structure on the surface (Figure 2a,d). These dendritic mesopores have wide surface channels and highly accessible surfaces, allowing for efficient functionalization of the DMSN surface with various species [32]. After the DMTP-TAPB COF growth, the surface of the DMSN was radically packed with closely intergrown nanoparticles (~10 nm in diameter) (Figure 2b,e). The DMSN@COF particle resembled a raspberry shape, and this shape was well-retained after removal of the DMSN template (Figure 2c,f), indicating that the replicated COF HP had a robust structure. The energy-dispersive X-ray (EDX) spectroscopy elemental mapping revealed that the C, N, and O co-existed in the particles, whereas the signal of Si was inappreciable, suggesting that the DMSN template had been completely removed (Figure 2g).

**Figure 3:**
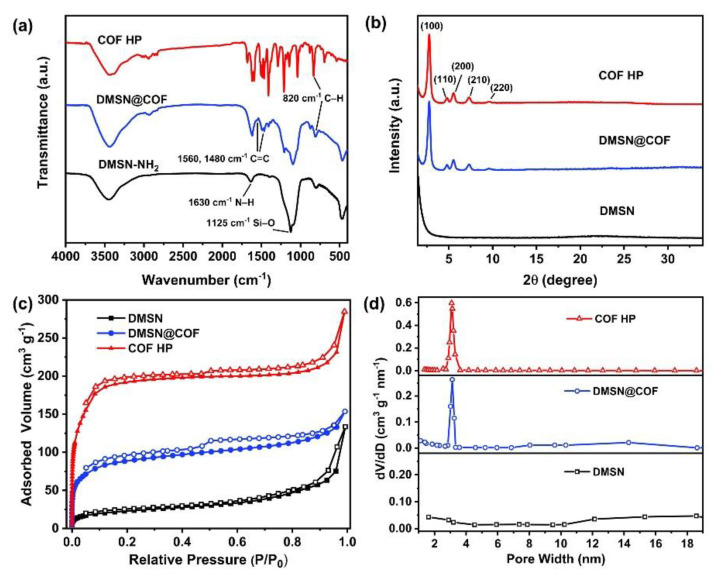
The FTIR spectra (**a**), XRD patterns (**b**), nitrogen adsorption–desorption isotherms (**c**), and pore size distribution (**d**) of DMSN, DMSN@COF, and COF HP.

The FTIR spectra were detected to monitor component changes of the samples (Figure 3a). The DMSN@COF particles exhibited the characteristic peaks of DMTP-TAPB COF such as the skeletal vibration peaks of C=C on the benzene ring at 1560 cm^−1^ and 1480 cm^−1^, and the out-of-plane bending vibration peaks at 820 cm^−1^, which are typical of substituted benzene-like C–H [19,45,46], verifying the formation of DMTP-TAPB COF in the DMSN template. These peaks were well-retained in the COF HP, confirming that the chemical structures of DMTP-TAPB COF remained intact after the removal of the DMSN template. In addition, the broad and strong peak at 1125 cm^−1^ was ascribed to the Si–O–Si stretching vibration [47]. This peak totally vanished in the COF HP, suggesting the efficient removal of the DMSN template.

The XRD patterns of DMSN, DMSN@COF, and COF HP are shown in Figure 3b. The DMSN only showed a weak and very broad peak at 20–30°, indicating its amorphous structure. In contrast, both the DMSN@COF and the COF HP showed prominent diffraction peaks at 3.2°, 5.5°, 6.1°, 7.3°, and 9.7°, which corresponded to the (100), (110), (200), (210), and (220) crystal planes of the DMTP-TAPB COF [19,45,46]. These data demonstrated the high crystallinity of the in situ grown DMTP-TAPB COF and the structural integrity of the DMTP-TAPB COF was well-persevered in the process for DMSN removal.

The surface area and porosity of the samples were determined from the N_2_ adsorption–desorption isotherms (Figure 3c). DMSN@COF and COF HP were discovered to have the type I nitrogen adsorption–desorption curves, which suggests their microporous structures [48]. As summarized in Table 1, the DMSN had the lowest Brunauer–Emmett–Teller (BET) surface area (276 m^2^/g). The surface area of the DMSN@COF was dramatically increased to 544 m^2^/g, which could be ascribed to the growth of a high surface area COF layer. After removing the DMSN template, the surface area of the COF HP was dramatically increased to 720 m^2^/g, outperforming the bulk DMTP-TAPB COF (608 m^2^/g) prepared in the absence of the DMSN template. The pore size distribution curves (Figure 3d) revealed that the DMSN@COF and COF HP had ordered micropores with a size centered at ~3.0 nm, in good agreement with the typical micropore size of DMTP-TAPB COF [19,45,46]. Notably, COF HP has a micropore volume of 0.52 cm^3^/g (Table 1), which was significantly higher than the micropore volumes of the hybrid DMSN@COF (0.27 cm^3^/g) and the bulk DMTP-TAPB COF (0.37 cm^3^/g).

**Figure 4:**
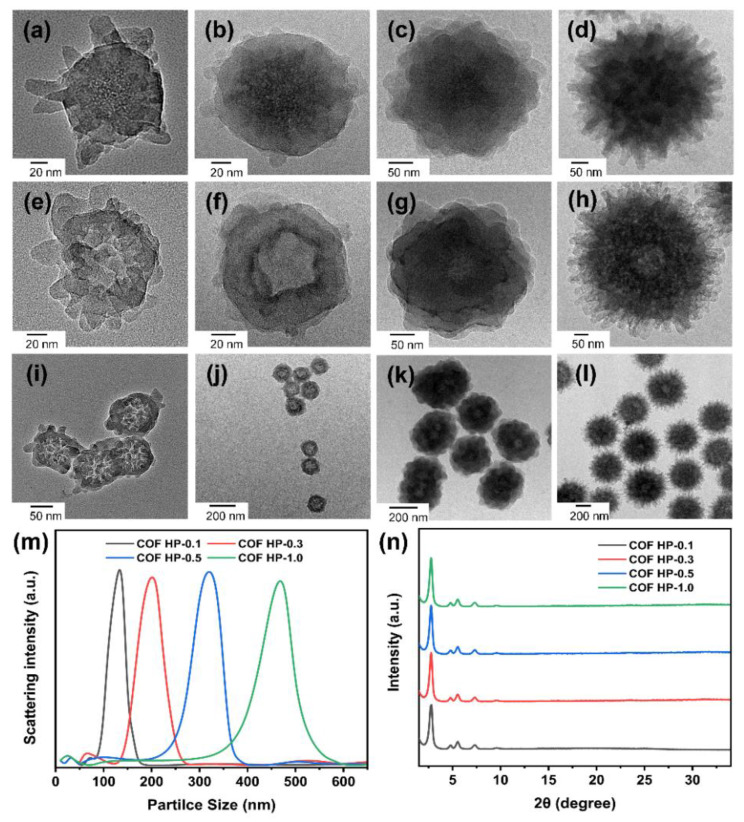
The TEM images of a series of DMSN@COF (**a**–**d**) and COF HP (**e**–**l**) synthesized with different COF and DMSN feed ratios: COF HP-0.1(**a**,**e**,**i**), COF HP-0.3 (**b**,**f**,**j**), COF HP-0.5 (**c**,**g**,**k**), and COF HP-1.0 (**d**,**h**,**l**). Particle size distributions (**m**) and the XRD patterns (**n**) of COF HP-0.1, COF HP-0.3, COF HP-0.5, and COF HP-1.0.

The thickness of the COF shell is tunable by changing the amounts of monomers (i.e., DMTP, TAPB) used in the COF growth. With the increase in the ratios of monomers to DMSN from 0.1 to 0.3, 0.5, and 1.0, the obtained DMSN@COF particle had a diameter of ca. 110, 150, 310, and 420 nm, respectively (Figure 4a–d). The COF layer was generally uniform, suggesting that the additional DMTP and TAPB could grow evenly on DMSN, and the thickness was controlled by the ratio of COF growth precursor molecules to DMSN in the feed ratio. The replicated COF HPs retained a similar particle size to the DMSN@COF and possessed a higher transparency (Figure 4e–l), confirming the cavity caused by the inorganic DMSN template. As plotted in Figure 4m and Table 2, the dynamic light scattering (DLS) data showed that when the COF monomer feeding ratio rose, the particle sizes increased from around 127 nm for COF HP-0.1 to 466 nm for COF HP-1.0. The XRD patterns in Figure 4n revealed that the COF HP of various thicknesses had identical diffraction peaks, indicating that the crystallinity of the COF HP did not vary with the different particle sizes.

**Figure 5:**
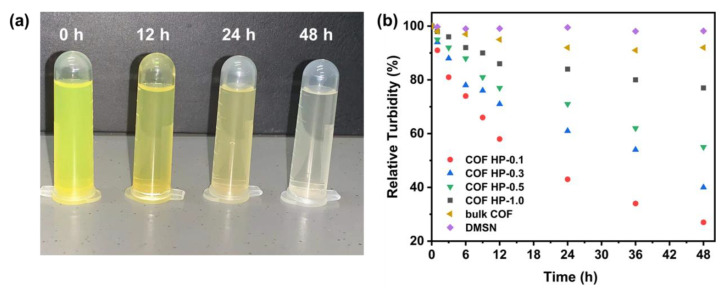
The optical photographs of the turbidity change of COF HP-0.1 in a pH 5.5 solution (**a**). The time-relative turbidity of bulk COF, DMSN, and COF HP of the various thicknesses in pH 5.5 solution (**b**).

Because the Schiff base structure of DMTP-TAPB COF possesses a reversible equilibrium, C=N is easily broken, especially in aqueous solutions under acidic conditions [49,50]. The COF HPs were stable under physiological conditions (37 °C, pH 7.4 PBS), however, the suspension of COF HP-0.1 gradually became transparent in 48 h (Figure 5a), indicating the deconstruction of the particles. The pH-labile degradation of COF HP was investigated by tracing the change in turbidity at pH 5.5 (Figure 5b), which is a typical microenvironment of tumor cells [51,52]. The degradation of the cleavable C=N bonds in DMTP-TAPB would lead to the COF HP deconstruction, and thereafter, the suspension of the turbidity decrease, which can be measured by DLS. As plotted in Figure 5b, COF HP-0.1 with the thinnest thickness had the fastest degradation. The relative turbidity of the COF HP-0.1 declined to ca. 50% and 23% after incubation for 12 h and 48 h, respectively, indicating a time dependent particle degradation manner. The degradation rate of COF HP decreased with increase in the COF layer thickness, however, it was significantly higher than that of the bulk COF, which still remained at a relative turbidity of 90% after 48 h. Although the C=N bonds in bulk COF can be hydrolyzed, the overall degradation of the bulk COF material is substantially slow due to the relatively stable π–π interaction between the COF layers. Due to their hierarchical pore structure, COF HP showed a more pH-labile deconstruction property.

Through intercalating with DNA, DOX has been widely used in the chemotherapy treatment of various types of tumors [53]. To reduce its cardiotoxicity side effects, an intracellular triggered release mechanism for the delivery of DOX is important. Due to the pH sensitivity and high affinity of DMTP-TAPB with DOX molecules [19], COF HPs were investigated for DOX delivery. To load DOX, the nanocarriers were incubated with DOX stock solution at room temperature for 24 h. As summarized in Table 1, the COF HP had a DOX loading of 46.8%, which was significantly higher than DMSN (15.2%), DMSN@COF (29.1%), and bulk COF (32.1%), and also the highest loading for DOX among the COFs available in the literature [16,17,18,19,21]. The extraordinary loading capacity of COF HPs were mainly attributed to the higher surface area and porosity of the materials (Table 1). In addition, no obvious difference in the loading was found for COF HP prepared with different thicknesses (Table 2).

**Figure 6:**
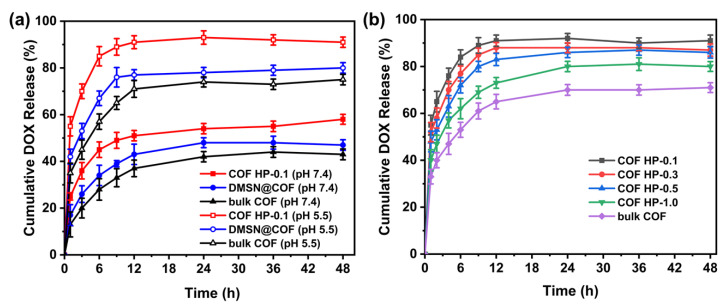
The DOX release profiles of bulk COF, DMSN@COF, and COF HP-0.1 in pH 5.5 and pH 7.4 solutions (**a**). The DOX release profiles of different series of COF HP (0.1, 0.3, 0.5, and 1.0) and bulk COF in the pH 5.5 solution (**b**).

Considering the bloodstream has a physiological pH of 7.4 and the subcellular compartments (i.e., endosomes, lysosomes) of tumor cells have an acidic condition of ca. pH 5.5 [54], two different pH conditions (pH 7.4 and 5.5) were used to study the in vitro release property of the DOX-loaded nanocarriers at 37 °C (Figure 6a). In general, the drug release was higher at pH 5.5 due to the cleavage of C=N bonds in DMTP-TAPB COF under acidic conditions, which caused the collapse of the polymer networks. Such release profiles are promising for intracellular drug delivery as the nanocarrier can be boosted, releasing its cargo under acidic conditions of the tumor cells. More importantly, the COF HPs showed a burst release (90% within the first 8 h), which was greater than the DOX released from DMSN@COF (~75%) and bulk COF (~55%). This can be attributed to the hierarchical pore structure and easier deconstruction properties of COF HPs. In addition, we evaluated the in vitro release of DOX from COF HP of various thicknesses (Figure 6b). The results showed that the COF HP-0.1 with the thinnest thickness had the fastest DOX release rate, which was in good agreement with the order of their degradation rates, as discussed in Figure 5b.

**Figure 7:**
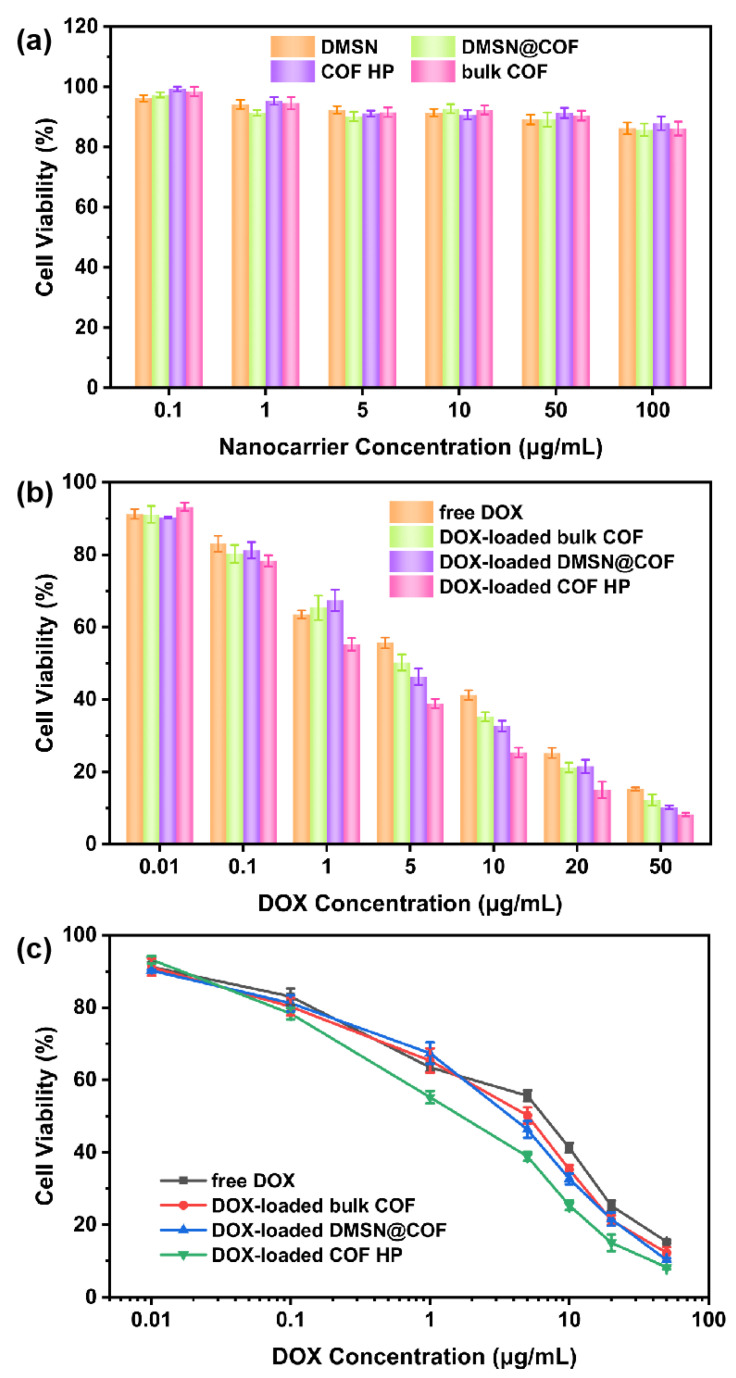
The biocompatibility of DMSN, bulk COF, DMSN@COF, and COF HP to the L929 cells (**a**). Cytotoxicity of the free DOX, DOX-loaded bulk COF, DMSN@COF, and COF HP to the A549 cells (**b**,**c**).

The biocompatibility of the nanocarriers was investigated by the standard MTT cell assays in the L929 cells (Figure 7a). For the MTT assay, the DMSN, DMSN@COF, COF HP and bulk COF were separately incubated with A549 cells at various particle concentrations (from 0.1 to 100 µg/mL) for 24 h. As shown in Figure 7a, all of the blank nanocarriers had a negligible effect on cell viability at particle concentrations of 0.01~10 µg/mL. An approximate 10% decrease in cell viability was found when the particle concentration was increased to 100 µg/mL, indicating that all of the nanocarriers had no obvious biological toxicity to the cells.

To assess the cytotoxicity of the DOX-loaded DMSN@COF, COF HP, and bulk COF, the in vitro cell viability tests against A549 cells were conducted. As shown in Figure 7b, the cell viability was reduced in a dose-responsive manner. The IC_50_ value of DOX-loaded COF HP was around 2 µg/mL (Figure 7c), which was lower than that of the DOX-loaded DMSN@COF (5 µg/mL), DOX-loaded bulk COF (6 µg/mL), and free DOX (9 µg/mL), respectively. The lowest IC_50_ value found in the DOX-loaded COF HP can most likely be attributed to the easier degradation and the faster drug release properties of COF HP in an acidic environment, as we have discussed before. These data demonstrate that the COF HP had considerable biocompatibility and is suitable for drug delivery.

## 4. Conclusions

In summary, we developed an efficient method for the preparation of COF HP by using the DMSN as a sacrificial template. The size of the COF HP could be easily tuned by adjusting the feeding concentrations of the monomers used for COF synthesis. The replicated COF HP had a surface area of 720 m^2^/g and a micropore volume of 0.52 cm^3^/g, outperforming the surface area (608 m^2^/g) and a micropore volume (0.37 cm^3^/g) of the bulk COF. The high porosity endowed the COF HP with excellent drug loading capacity (46.8 wt% for DOX), the highest loading of DOX in COF materials in the literature. Notably, the COF HP had a COF layer thickness-dependent degradation property. The COF HP-0.1 had the fastest degradation rate, and 55% of the particles could be degraded at pH 5.5 in 24 h. The COF HP also had a pH-responsive drug release behavior. A burst release (ca. 90% within the first 8 h) was found in the COF HP, which was much faster than the DOX released from the bulk COF (~55%). The in vitro cell experiments demonstrated that the IC_50_ value of the DOX loaded in COF HP was 2–3 times lower than that of the DOX loaded in the bulk COF and the hybrid DMSN@COF. Attributed to the high loading capacity and tailored particle deconstruction properties, the COF HPs showed great potential in its application as drug delivery vehicles. Considering the dynamic research area of COFs, we believe that the strategy developed in this work is also applicable to preparing various types of COF HPs with rationally designed properties for applications in molecular separation and catalysis.

## Figures and Tables

**Table 1:** The texture properties and DOX loading capacity of different materials.

Sample	DMSN	DMSN@COF	COF HP	Bulk COF
Surface area (m^2^/g)	276.3	544.1	720.7	608.2
Micropore volume (cm^3^/g)	-	0.266	0.523	0.371
DOX loading (*w*/*w*, %)	15.2	29.1	46.8	32.1

**Table 2:** The particle size, zeta potential, and DOX loading capacity of COF HP-0.1, COF HP-0.3, COF HP-0.5, and COF HP-1.

Sample	COF HP-0.1	COF HP-0.3	COF HP-0.5	COF HP-1
Particle size (nm)	127 ± 14	197 ± 10	317 ± 23	466 ± 28
Zeta potential (mV)	+15.2	+15.8	+15.5	+14.7
DOX loading (*w*/*w*, %)	46.8	44.5	41.7	40.3

## Data Availability

Not applicable.
